# Trends in human papillomavirus (HPV) infection among HIV-positive women in the pre-HAART and HAART era in a Nigerian clinic

**DOI:** 10.1186/1758-2652-13-S4-P217

**Published:** 2010-11-08

**Authors:** MO Onigbogi, OY Ojo

**Affiliations:** 1 Lagos University Teaching Hospital, Community Medicine, Lagos, Nigeria

## Background

The prevalence of HIV infection has been on the increase in Nigeria in recent times. HIV-positive patients frequently have anogenital malignancies due to HPV. HAART was introduced in Anti-retroviral (ARV) centers in Nigeria in the year 2002.

## Purpose of the study

To determine trends in incidence of anogenital malignancies among HIV-positive women undergoing treatment in the clinic in the pre-HAART and HAART era.

## Methods

A retrospective review of 541 case notes of HIV-positive female patients from January 1999 to December 2004 were analyzed by utilizing an on-going observational database at the ARV center. Rate ratios, comparing incidence rates (number of malignancies per 1000 person years) were calculated.

## Results

Twenty-four (4.43%) of the patients had one form of anogenital manifestation of HPV or the other. The incidence rate for HPV rose from 2.28 in the pre-HAART era to 6.40 in the HAART era (Rate ratio = 3.15; 95% confidence interval (CI) =1.31 - 7.44; p= 0.0002).

## Conclusions

There has been a significant rise in the incidence of HPV since the introduction of HAART. This may be due to the longer survival of HIV-infected patients, surpassing the latency period for the anogenital malignancies. Care providers should be more vigilant for HIV-associated malignancies as patients live longer in this part of the world.

